# Lipid Mediator Informatics and Proteomics in Inflammation-Resolution

**DOI:** 10.1100/tsw.2006.118

**Published:** 2006-06-01

**Authors:** Yan Lu, Song Hong, Katherine Gotlinger, Charles Serhan

**Affiliations:** Center for Experimental Therapeutics and Reperfusion Injury, Department of Anesthesiology, Perioperative and Pain Medicine, Brigham and Women's Hospital Harvard Medical School, 75 Francis Street, Boston, MA 02115, USA

**Keywords:** leukocytes, mapping, mass spectrometry, ω-3 PUFA, lipoxins, eicosanoids, resolvins, protectins

## Abstract

Lipid mediator informatics is an emerging area denoted to the identification of bioactive lipid mediators (LMs) and their biosynthetic profiles and pathways. LM informatics and proteomics applied to inflammation, systems tissues research provides a powerful means of uncovering key biomarkers for novel processes in health and disease. By incorporating them with system biology analysis, we review here our initial steps toward elucidating relationships among a range of bimolecular classes and provide an appreciation of their roles and activities in the pathophysiology of disease. LM informatics employing liquid chromatography-ultraviolet-tandem mass spectrometry (LC-UV-MS/MS), gas chromatography-mass spectrometry (GC-MS), computer-based automated systems equipped with databases and novel searching algorithms, and enzyme-linked immunosorbent assay (ELISA) to evaluate and profile temporal and spatial production of mediators combined with proteomics at defined points during experimental inflammation and its resolution enable us to identify novel mediators in resolution. The automated system including databases and searching algorithms is crucial for prompt and accurate analysis of these lipid mediators biosynthesized from precursor polyunsaturated fatty acids such as eicosanoids, resolvins, and neuroprotectins, which play key roles in human physiology and many prevalent diseases, especially those related to inflammation. This review presents detailed protocols used in our lab for LM informatics and proteomics using LC-UV-MS/MS, GC-MS, ELISA, novel databases and searching algorithms, and 2-dimensional gel electrophoresis and LC-nanospray-MS/MS peptide mapping.

## INTRODUCTION

To qualify as a lipid mediator (LM), a product must be stereoselective in its actions and be generated by cells in quantities that are commensurate with its potency and range of action[1].

*Yan Lu and Song Hong share first authorship, contributing equally to this manuscript.
**Corresponding author.

"Lipid mediator informatics" is the term we use for the new area of identifying bioactive lipid mediators (LMs) and their biosynthetic profiles and pathways using a computer-based automated system equipped with databases and novel searching algorithms. This will provide the foundation for the use of specific LMs as markers in therapeutic interventions[2]. As currently practiced, lipidomics can be subdivided into architecture/membrane-lipidomics and mediator-lipidomics. The mapping of structural components and their relation to cell activation, as well as generation of potent LMs and networks, involved a mass spectrometry-computational approach[2] to appreciate the inter-relationships and complex mediator networks important for cell homeostasis.

Advances in computers, software, algorithms, chromatography, and identification of bioactive mediators help to form the basis of our appreciation for mediator profiling[3]. Advances in the use of liquid chromatography-tandem mass spectrometry (LC-MS/MS) permit profiling of closely related compounds. Moreover, the interface of MS/MS with LC permits profiling of lipid-derived mediators with reduced potential for work-up induced artifacts. Liquid chromatography-ultraviolet spectrometry-tandem mass spectrometry instrumentation (LC-UV-MS/MS) is the most reliable and sensitive method to identify and quantify these LMs[4,5]. Routine lipidomic analyses are outlined in Scheme 1. (A complete list of terms and abbreviations used in this report can be found at the end of the text.) Databases with appropriate search algorithms are crucial for the timely identification of these LMs via LC-UV-MS/MS analytical runs[2].

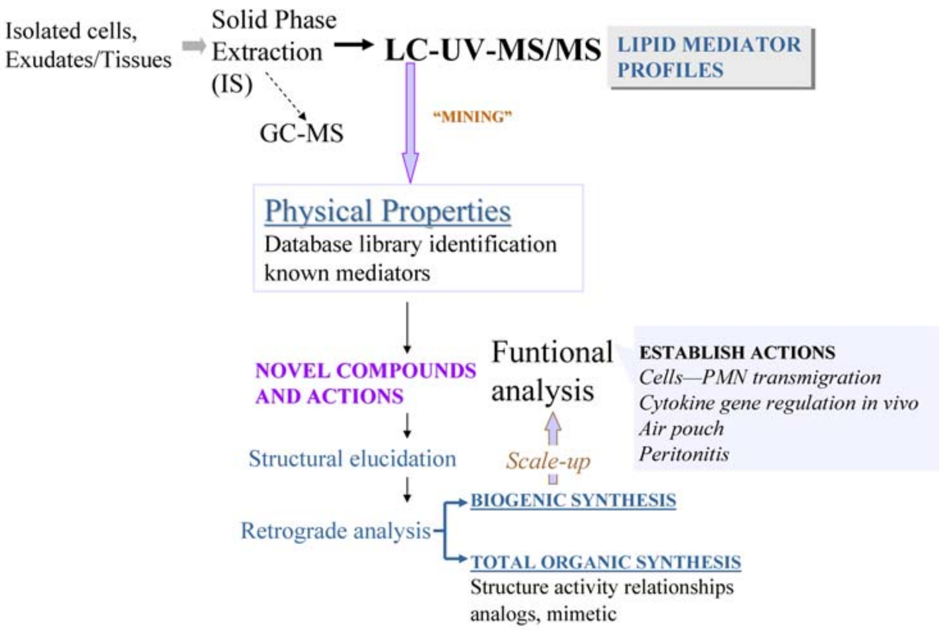

**SCHEME 1.** Mediator lipidomics via LC-UV-MS/MS analysis of the physicochemical properties including tandem mass and UV spectra and chromatographic profiles.

Low-energy ionization with electrospray avoids unwanted degradation and generates primarily molecular (or pseudomolecular) ions for collision-activated dissociation MS/MS analysis[4]. LC-UV-MS/MS can provide more direct spectral characterization for structural elucidation than GC-MS (gas chromatography coupled with MS) because samples can be analyzed without prior derivatization. The correlation of MS/MS fragments vs. structures of some LMs and their isomers has been determined[4,6,7,8]. The results indicate that physical properties are readily obtained and used for complete structural elucidation of LMs.

GC-MS is also useful to provide additional information together with LC-UV-MS/MS to support structural identification and proposed structures. LC-UV is a widely used technique for eicosanoid analysis[9]. ELISA (enzyme-linked immunosorbent assay) is designed for quantification of specific LMs with high selectivity and sensitivity. It allows investigators to analyze a large number of samples in a timely fashion[10].

"Proteomics" is the study of the major functional component of the genome, i.e., the identification of all proteins in a chosen biological system and all their post-translational modifications[11]. This approach will be used to characterize genes and functional interactions among proteins that are important in inflammation and allow detection of subtle differences in protein levels that provide a detailed picture of inflammation.

Separation of proteins by two-dimensional (2D) gel electrophoresis coupled with identification of proteins by tryptic peptide capillary LC-nanoelectrospray ionization (nanospray) ion-trap MS/MS followed by protein database searching using MS/MS spectra is a powerful analytical method in proteomics as depicted in Scheme 2.

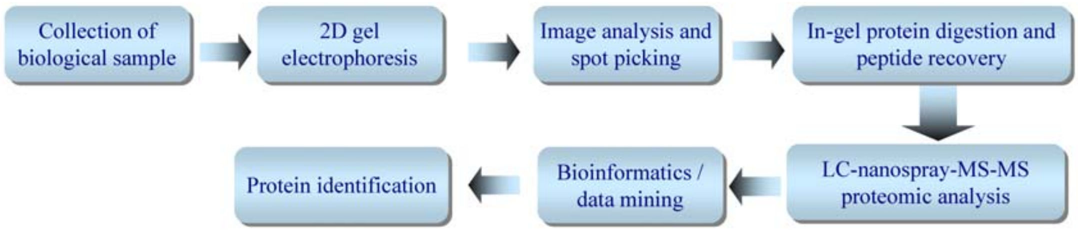

**SCHEME 2.** Proteomics: 2D gel electrophoresis and LC-nanospray-MS/MS-based analysis.

The comparison of changes in intensity and mobility of proteins of interest on 2D gels between samples from different treatment groups translates directly to changes in protein expression and modification of primary structure. Through identification of sets of proteins that are concertedly up- or down-regulated, the dynamic changes following a specific stimulus can be charted[11].

## NOVEL LIPID MEDIATOR PATHWAYS IN INFLAMMATION AND RESOLUTION

It is now appreciated that inflammation plays a key role in many prevalent diseases. In addition to the chronic inflammatory diseases such as arthritis, psoriasis, and periodontitis, it is now increasingly apparent that diseases such as asthma, Alzheimer's disease, and even cancer have an inflammatory component associated with the disease process. Therefore, it is important for us to gain more detailed information on the molecules and mechanisms controlling inflammation and its resolution[12]. Toward this end, we recently identified new families of LMs generated from fatty acids during resolution of inflammation, termed resolvins and protectins[8,13,14] (Fig. 1) (Table 1).

Resolvins and protectins are autacoids that play a critical and broad role in human health and diseases, especially those related to inflammation and resolution[3]. These novel mediators generated from eicosapentaenoic acid (EPA) and docosahexaenoic acid (DHA) that displayed potent bioactions were first identified in resolving inflammatory exudates and in tissues enriched with DHA[7,8,13]. The trivial names resolvin (resolution phase interaction products) and docosatrienes (DTs) were introduced for the bioactive compounds belonging to these novel series because they demonstrate potent anti-inflammatory and immunoregulatory actions. The compounds derived from EPA carrying potent biological actions (i.e., 1–10 nM range) are designated E series, given their EPA precursor, and denoted as resolvins of the E series (resolvin E1 or RvE1), and those biosynthesized from the precursor DHA are resolvins of the D series (resolvin D1 or RvD1). Bioactive members from DHA with conjugated triene structures are DTs that are immunoregulatory[8,13] and neuroprotective[15] and are termed protectins/neuroprotectins. Aspirin

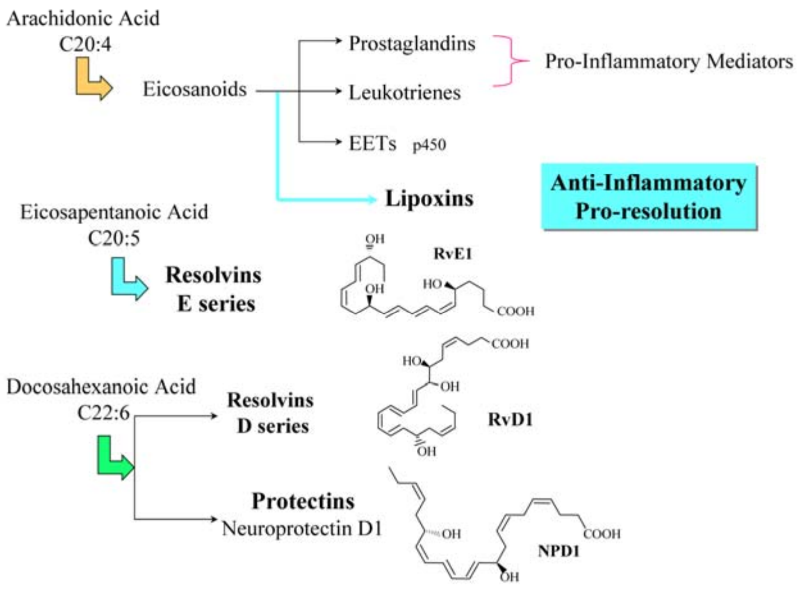

**A**

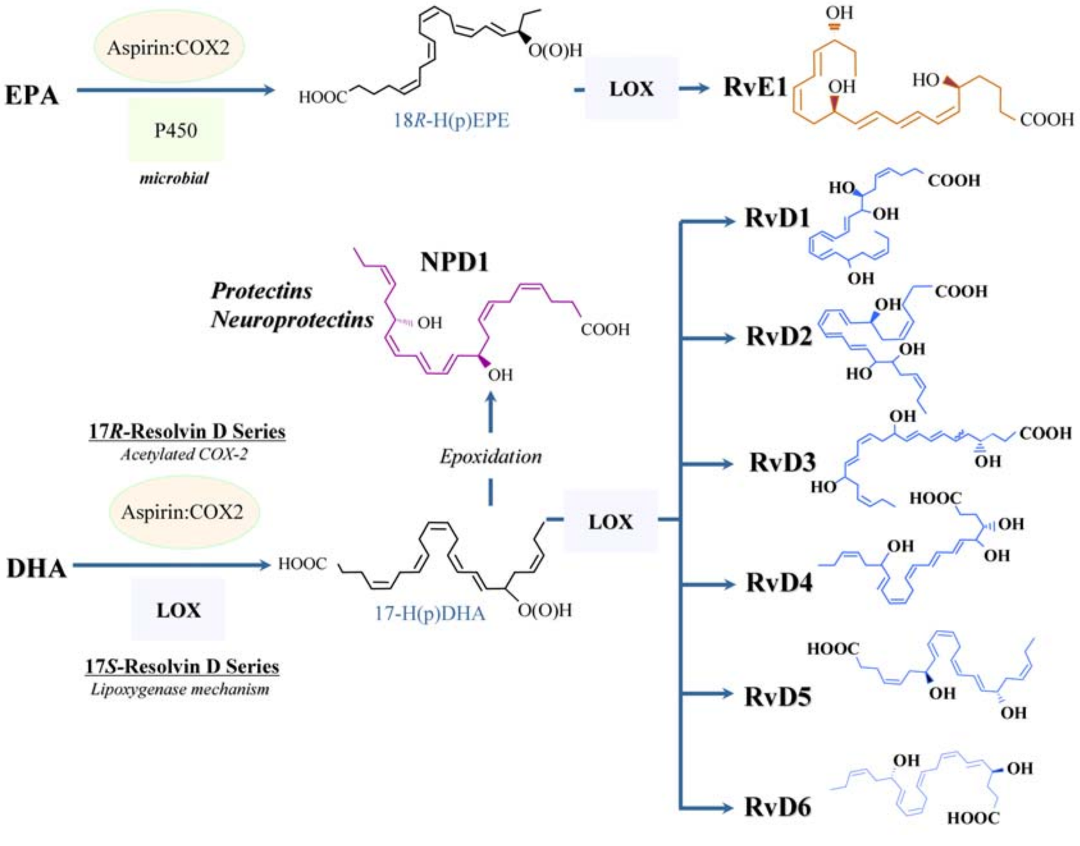

**B**

**FIGURE 1.** (A) Families of bioactive lipid autacoids. Arachidonic acid is the precursor for many of the known bioactive mediators, epoxyeicosatrienoic acids (EETs), prostaglandins, leukotrienes, and lipoxins (both pro- and anti-inflammatory mediators). The omega-3 (polyunsaturated fatty acids) PUFA, EPA (C22:5) and DHA (C22:6), are precursors to potent new families of mediators termed resolvins and protectins (see text for details). (B) Formation of resolvin E1 derived from EPA, resolvin D1, protectins, and neuroprotectin D1 from DHA.

## TABLE 1

### Resolvins and Protectins: Bioactions in Animal Disease Models and Formation in Cells, Tissues and Organs

| Mediator | Bioactions | Reference | Formation in Cells, tissues, and organs | Reference |
|---|---|---|---|---|
| Resolvin E1 | Reduces PMN infiltration, murine skin air pouch inflammation, peritonitis | Serhan et al., J. Exp. Med. 2000[7];<br>Serhan et al., J. Exp. Med. 2002[8];<br>Arita et al. J. Exp. Med. 2005[40];<br>Bannenberg et al., J. Immunol. 2005[37] | Murine dorsal porch Inflammatory exudates;<br><br>Periodontitis exudate;<br><br>Human Plasma<br><br>Trout brain | Serhan et al., J. Exp. Med. 2000 [7];<br>Arita et al., PNAS 2005[41]<br>Arita et al. J. Exp. Med. 2005 [40];<br><br>Lu et al. in submission[42] |
| | Gastrointestinal protection in TNBS colitis | Arita et al., PNAS 2005[41] | | |
| | Protects in periotonitis, stops inflammation and bone loss | Hasturk et al., FASEB J. 2005[43] | | |
| Resolvin D1 | Reduces PMN infiltration, murine skin air pouch inflammation | Serhan et al., J. Exp. Med. 2002[8] | Human PMN;<br><br>Ischemic-injury kidney | Hong, et al. in submission[44];<br>Hong, et al. 54th ASMS comference[45]<br>Duffield et al., submitted[46] |
| | Reduces peritonitis<br>Reduces cytokine expression in microglial cells | Hong et al., J. Biol. Chem. 2003[13] | | |
| | Protects in renal ischemic injury | Duffield et al., submitted[46] | | |
| AT-RvD1 | | | Murine peritonitis exudates;<br><br>Murine brain stroke | Serhan et al., J. Exp. Med. 2002 [8];<br>Marcheselli et al., J. Biol. Chem. 2003[15] |
| AT-RvD2 to RvD6 | | | Murine dorsal airpouch exudates | Serhan et al., J. Exp. Med. 2002 [8]; |
| RvD2 | | | Human whole blood;<br><br>Ischemic-injury kidney<br>Trout brain | Hong et al., J. Biol. Chem. 2003[13]<br>Duffield et al., submitted[46]<br>Hong et. al. 2005 [38] |
| RvD4 | Reduce ischemic injury of kidney | Duffield et al., submitted[46] | Human whole blood;<br><br>Ischemic-injury kidney | Hong et al., J. Biol. Chem. 2003[13];<br>Duffield et al., submitted[46] |
| RvD5 | Reduce PMN infiltration | Hong et al., J. Biol. Chem. 2003[13] | Human whole blood;<br>Human glial cells;<br>Ischemic-injury kidney<br>Trout brain | Hong et al., J. Biol. Chem. 2003[13]<br>Duffield et al., submitted[46]<br>Hong et. al. 2005 [38] |
| RvD6 | Reduce PMN infiltration | Marcheselli et al., J. Biol. Chem. 2003[15] | Human whole blood;<br>Human PMN;<br>Human glial cells;<br>Ischemic-injury kidney | Hong et al., J. Biol. Chem. 2003[13]<br>Duffield et al., submitted[46] |

| | | | | |
|---|---|---|---|---|
| Omega-22-hydroxy-RvD6 | | | Ipsilateral mouse hippocampus during reperfusion following transient ischemia | Marcheselli et al., J. Biol. Chem. 2003[15] |
| Protectin D1/ neuroprotectin D1 | Reduces stroke damage | Marcheselli et al., J. Biol. Chem. 2003[15] | Human whole blood; Human PMN and its ω-22 oxidation product;<br>Human glial cells;<br>Murine brain stroke;<br>Human retinal pigment epithelium<br>Ischemic-injury kidney Trout brain | Hong et al., J. Biol. Chem. 200[13]; Marcheselli et al., J. Biol. Chem. 2003[15]; Mukherjee et al., PNAS 2004[47]; Duffield et al., submitted[46] Hong et. al. 2005 [38] |
| | Reduces PMN infiltration | Hong et al., J. Biol. Chem. 2003[13] | | |
| | Protects from retinal injury | Mukherjee et al., PNAS 2004[47] | | |
| | Regulates $T_H2$ cells, apoptosis and raft formation | Ariel et al., J. Biol. Chem. 2005[48] | | |
| | Shortens resolution interval in murine peritonitis; regulates cytokines and chemokines | Bannenberg et al., J. Immunol. 2005[37] | | |
| | Reduces PMN infiltration; reduces peritonitis | Serhan et al. 2006[39] | | |
| | Diminished production in human Alzheimer disease and promotes neural cell survival | Lukiw et al., J. Clin. Invest. 2005[49] | | |
| | Promotes corneal epithelial cell wound healing | Gronert et al., J. Biol. Chem. 2005[50] | | |

treatment impacts biosynthesis of these compounds and a related series by triggering endogenous formation of the 17*R*-D series resolvins and docosatrienes. These novel epimers are denoted as aspirin-triggered (AT)-RvDs and -DTs, and possess potent anti-inflammatory actions *in vivo* essentially equivalent to their 17*S* series pathway products.

## LIPID MEDIATOR INFORMATICS

### Criteria for Identification of a Bioactive Lipid Mediator

The criteria to identify a known LM for LC-UV-MS/MS-based lipidomic analysis are as follows[9,16]:

1. LC retention time should match by coelution with the LM authentic standard.
2. UV chromophore should match the LM standard (i.e., λmax and band shape).
3. MS/MS spectrum should have the fragment ions of (M-H), (M-H-$CO_2$), (M-H-n$H_2O$) (n is the number of hydroxyl groups in the LM) and ions generated from at least one of two cleavages on the bonds directly linked to the carbon of the function group.

The criteria to identify an LM for standardized GC-MS-based lipidomic analysis are as follows:

1. The GC retention time should match by coelution with the LM standard.
2. GC-MS spectrum of unknown should match the standard spectrum (at least six ions).

## Sample Extraction Procedures for Lipidomic Analysis

All incubations and *in vivo* samples (i.e., exudates and tissues) will be stopped with 2 vol of cold methanol[15]. Briefly, this procedure is tailored as follows: an internal standard [for example, 50 ng of a deuterium-labeled LM (e.g., $d_4$-$PGE_2$ or $d_4$-$LTB_4$) or $PGB_2$] will be selected and added to determine the extraction recovery (typically >90%) of the LMs. The samples are centrifuged (3,000 rpm, 4°C, 15 min) to remove cellular and protein materials. After the supernatants are decanted, they are diluted with 5 vol of Milli-Q water. The pH is adjusted to 3.5 with 1 *M* HCl for C18 solid-phase extraction (SPE). After washing with 15 ml of $H_2O$ and then 8 ml of hexane, the SPE cartridges (C18, 3 ml, Waters, MA) are eluted with 8 ml of methyl formate, and the effluent is reconstituted into methanol for lipidomic analysis using LC, GC-MS, or LC-MS/MS[17,18].

## GC-MS-Based Lipidomic Analysis

These LMs need to be converted to derivatives for GC-MS analysis. The derivatization includes methylation of carboxyl groups and silylation of hydroxyl groups to trimethyl-siloxy groups. Methylation will be conducted *via* reaction with diazomethane. MNNG (1-methyl-3-nitro-1-1nitroguanidine, N-methyl-N'-nitro-N-nitrosoguanidinine) (Sigma-Aldrich, MO) is converted with 5 *N* NaOH solution to diazomethane gas, which is trapped into ice-cold diethyl ether. Each sample will be treated in 0.5 ml of ether solution of diazomethane for 30 min at room temperature. After the ether and extra diazomethane are removed with $N_2$, the sample will be silylated with 0.1 ml of BSTFA (N,O-bis[trimethylsilyl]trifluoro-acetamide) reagent (Pierce, IL) for 24 h and protected from light as in Serhan[19]. GC-MS will be performed with an Agilent GC 6890 gas chromatograph coupled with a 5973 MS mass spectrometer (Agilent Technologies, CA). The conditions typically are column, HP-1 0.25 mm × 0.25 μm × 30 m (Agilent); splitless on time, 0.9 min; column temperature program, 150ºC (1 min), 230ºC (4 min), 240ºC (8 min), and 245ºC (12 min) as in Nicolaou et al.[16] and Serhan[19].

## LC-UV-Based Lipidomic and Chiral Analysis

LC-UV-based lipidomic analysis of LMs will be conducted on an Agilent 1100 HPLC-UV system or an Agilent 1040 HPLC-UV system with the photodiode-array (PDA) UV detector scanned from 200–360 nm. The general conditions are as follows for achiral LC-UV, a Prodigy ODS (3) (100 x 2 mm x 5 mm) column (Phenomenex, CA) will be used. The mobile phase runs at 0.2 ml/min as C (methanol:water:acetic acid 65:34.99:0.01) from 0–8 min, ramps to methanol from 8.01–30 min, then flows as methanol for 5 min, and then runs as C again. For chiral LC-UV, a Chiralcel OB-H column (4.6 × 250 mm) (Chiral Technologies, PA) will be used to determine *R* and *S* alcohol configurations of monohydroxy-PUFA using isocratic mobile phase (hexane:isopropanol 95:5), with a 0.6 ml/min flow rate. The LM analytes are methylated before chiral LC analysis. The identification will be conducted by matching the retention times and UV spectra of unknown compounds to standards. After the compounds of interest are identified, they will be quantified on the basis of their chromatographic peak areas and calibration curves of chromatographic peak areas for authentic synthetic standards[9].

## LC-UV-MS/MS-Based Lipidomic Profiling

LM informatics analysis will be conducted on a LCQ™ LC-PDA-ion trap-MS/MS (ThermoFinnigan, CA) equipped with a LUNA C18-2 (150 x 2 mm x 5 mm) or Prodigy ODS (3) (100 x 2 mm x 5 mm) column (Phenomenex, CA) with photodiode-array UV detector scans from 200–360 nm. The conditions are as follows: the mobile phase flows at 0.2 ml/min as C (methanol:water:acetic acid 65:34.99:0.01) from 0–8

min, ramps to methanol from 8.01–30 min, then flows as methanol for 10 min, and then runs as C again for 10 min. Conditions for MS/MS are electrospray voltage, 4.3 kV; heating capillary, –39 V; tube lens offset, 60 V; sheath $N_2$ gas, 1.2 l/min; and auxiliary $N_2$ gas, 0.045 l/min[20].

Quantification will be based on the peak areas from selective ion monitoring (SIM) chromatograms and the calibration curve of chromatographic areas for each corresponding standard. Examples of LC-UV-MS/MS-based lipidomic analysis of eicosanoid standards are shown in Fig. 2. Fig. 3 shows the chromatograms of biogenic resolvins, AT-Rvs, and PD1/NPD1. Fig. 4 displays the MS/MS spectra of biogenic resolvins, AT-Rvs, and 17*S*-HDHA. Mass spectra of biogenic and synthetic PD1/NPD1 are given in Fig. 5; MS/MS spectrum of synthetic RvE1 and GC-MS spectrum of deuterated-RvE1 are shown in Fig. 6.

## *Lipidomic Databases and Searching Algorithms*

Using current chemical analytical technologies, most LMs are identified manually by direct comparison of the spectra, chromatographic behaviors, and in some cases biological activities acquired from sample tissues with those of authentic standards of known LMs; when authentic standards are not available, as in the case of novel LMs and their further metabolites, basic chemical structures can be obtained on the basis of the relationship between structures and features of their spectra and chromatographic behaviors compared to those of synthetic and biogenic products prepared to assist in the assignment. We routinely identify LMs by matching the unknown spectra (MS/MS, GC-MS, and UV spectra) and retention times (RTs) to those of authentic and synthetic standards if available, or with a theoretical database that consists of virtual UV and MS/MS spectra and RTs for discovering potentially novel LMs[2,8,13] if standards are not available. We initially developed a theoretical database and algorithm according to the relationships between LM structures and their spectral and chromatographic characteristics[2]. The proposed structures of novel potential LMs in the theoretical databases were based on PUFA precursors and established biosynthetic pathways.

Mediator-lipidomic databases and search algorithms were constructed to assist in the identification of LM structures employing LC-UV-ion trap MS/MS with the following objectives: (1) assembling a database using currently available mass-spectral software, (2) constructing a cognoscitive-contrast-angle algorithm and databases to improve the identification of LMs using MS/MS ion identities that currently cannot be performed with available software, and (3) developing a theoretical database and algorithm for assessing potentially novel and/or unknown structures of LMs and their further metabolites in biologic matrices. It is quite meaningful to develop mediator-lipidomic databases and algorithms using ion trap mass spectrometers that are relatively cheaper and popular. Moreover, the fragmentation rules and patterns for collision-induced dissociation (CID) spectra from triple-quadruple mass spectrometers, another popular MS instrumentation, are similar to what we encounter using ion trap[6,21].

### Logic Diagram to Identify Lipid Mediators

The regular routes for LM identification and structure elucidation of potentially novel LMs were followed in mediator-lipidomic databases and search algorithms that we constructed (Scheme 3). Two types of lipidomic databases for LMs were used for searching; one contains LC-UV-MS/MS spectra and chromatograms acquired on LM standards and the other is based on theoretically generated LC-UV-MS/MS

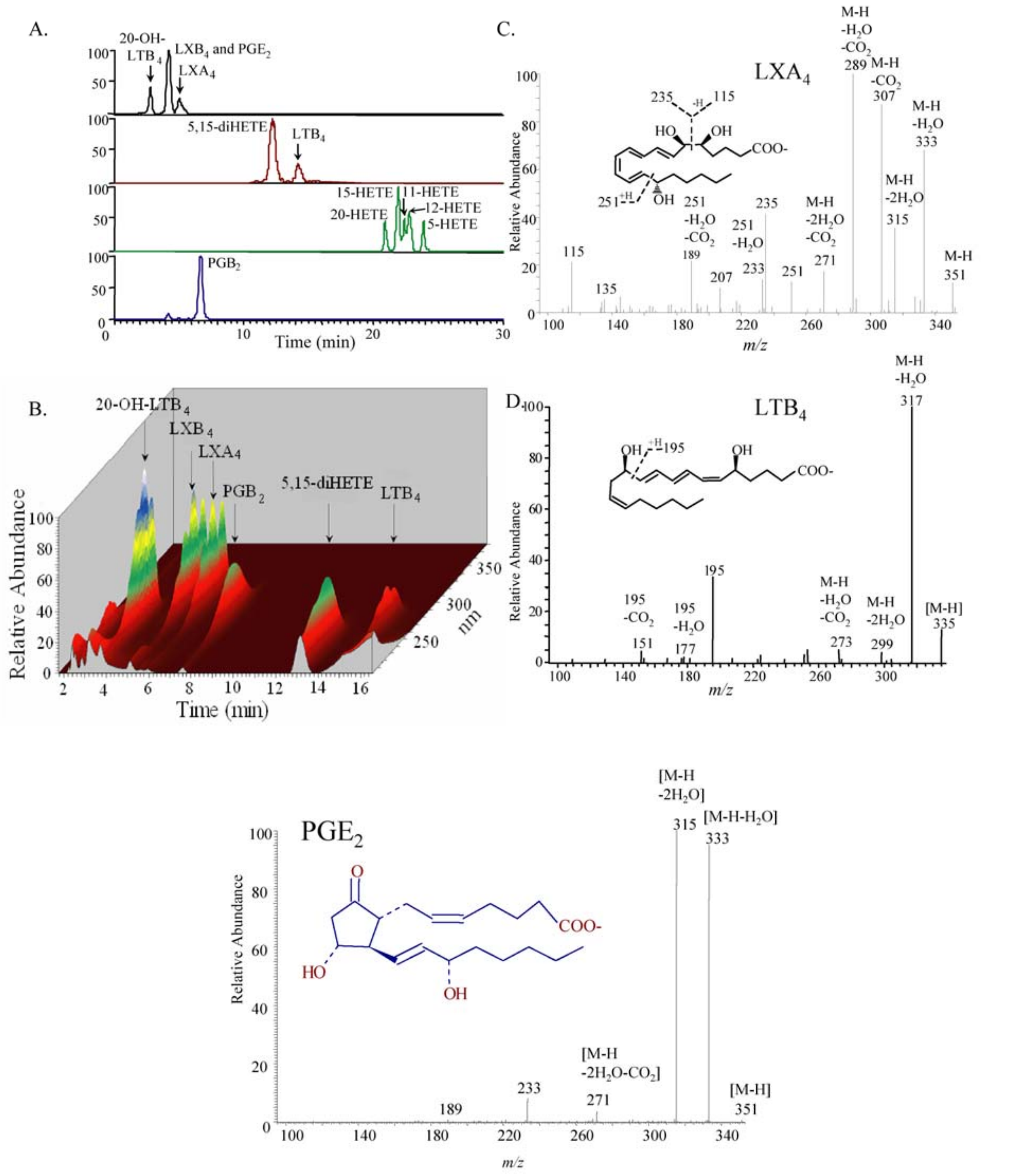

E

**FIGURE 2.** LC-UV-MS/MS-based lipidomics for eicosanoids biosynthesized via LOX and COX pathways. Analysis was conducted using LC-UV-MS/MS. (A) SIM chromatograms showing triHETEs and PGE2 (m/z 351), diHETEs (m/z 335), and HETEs (m/z 319). (B) The 3-dimensional display of LC-UV spectra and chromatograms showing maximal absorbance at 300 nm for $LXA_4$ and $LXB_4$, at 270 nm for 20-hydroxy-$LTB_4$ and $LTB_4$, and at 240 nm for 5,15-diHETE. (C) MS/MS spectrum (m/z 351) for $LXA_4$. (D) MS/MS spectrum (m/z 335) for $LTB_4$. (E) MS/MS spectrum (m/z 351) for $PGE_2$.

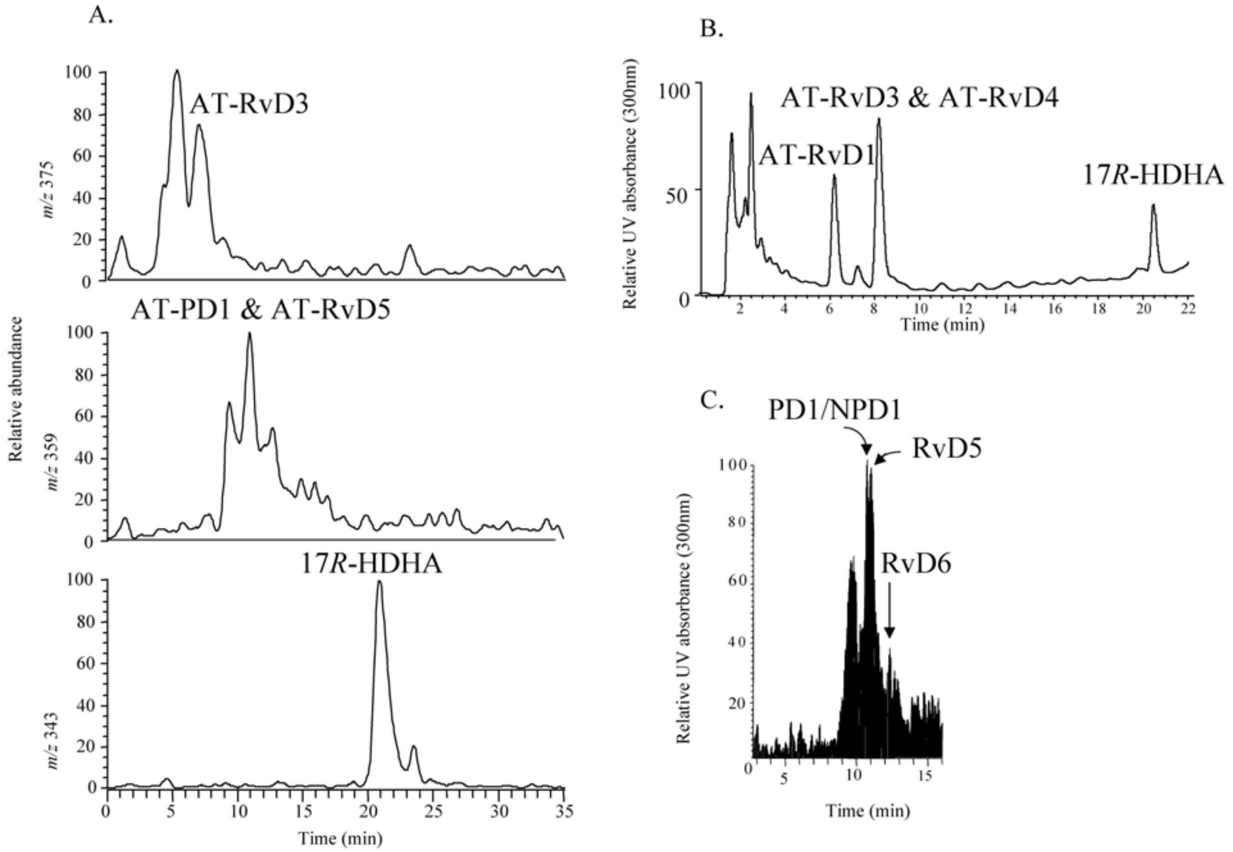

**FIGURE 3.** Chromatograms of biogenic resolvins, AT-Rv, and protectin D1. (A and B) AT inflammatory exudates produce 17*R*-containing AT-Rvs. Exudates were obtained and analyzed by procedures essentially identical to those described in Murphy et al.[6]. Selective ion chromatograms were at m/z 375 (top), 359 (middle), and 343 (bottom). The UV chromatogram was plotted at 300 nm to mark tetraene-containing chromatophores. (C) Selected ion chromatogram (m/z = 359) shows 17*S* series resolvins and protectins produced in human neutrophils ($30–50 \times 10^6$ cells/incubation), which were exposed to zymosan A and 17*S*-H(p)DHA, and products were analyzed using LC-UV-MS/MS (n = 5)[13].

spectra and chromatograms. The searches were conducted stepwise against either standards or the theoretical databases to increase the search speed. The search of MS/MS spectra was carried out only against the MS/MS subdatabase with the molecular ion of interest (i.e., M-1) and matched UV spectra (e.g., conjugated diene, triene, or tetraene chromophores). Subsequently, the matching of RTs was performed. If the UV spectral pattern was unclear, the MS/MS and RT were still searched to avoid potential errors in assignment. A standard LM or theoretical fragmentation/ion fragmentation pattern that fulfilled the above match criteria was then assigned to the unknown set. If the match was a "hit" only with UV and MS/MS spectra, but not with RT, the LM in the sample was likely to be a geometric isomer of a known LM.

## Databases Constructed with MassFrontier$^{TM}$ Software

A mediator-lipidomic database composed of LC-UV-MS/MS spectra and chromatograms acquired from authentic LMs was constructed with GC-MS spectral software MassFrontier$^{TM}$ (ThermoFinnigan). The search algorithm for MassFrontier$^{TM}$ is dot-product, developed by Stein et al.[22,23,24]. The UV $\lambda_{max}$ of authentic LMs were written into the subdatabase names and the RTs were written into the LM names so that MassFrontier™ could handle the acquired UV spectral results and RTs for the identification of the unknown LMs following the logic diagram in Scheme 3.

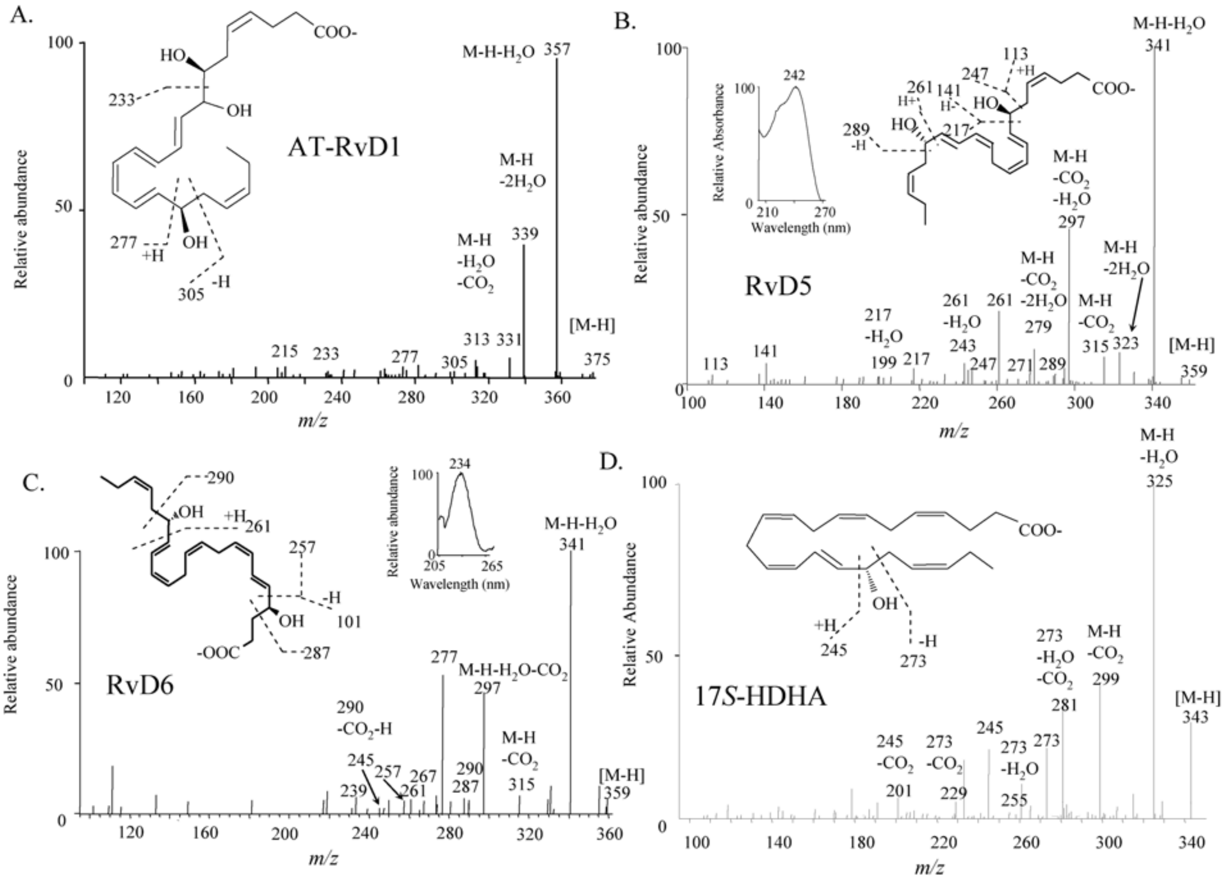

**FIGURE 4.** MS/MS spectra of biogenic resolvins, AT-Rv, and 17*S*-HDHA. (A) AT-RvD1 (7*S*, 8,17*R*-triHDHA) was generated in AT inflammatory exudates[3]. (B) RvD5 was generated in trout brain cells[38]. (C) RvD6 was produced by human PMN; (inset) UV spectrum[13]. (D) 17*S*-DHA was generated in trout brain[38].

## COCAD: Cognoscitive-Contrast-Angle Algorithm and Databases

The system with **c**ognoscitive-**c**ontrast-angle **a**lgorithm and **d**atabases (COCAD) that we developed can be used to elucidate the fragmentation of LMs in mass spectrometry and to match unknown MS/MS spectra to those of synthetic and/or authentic standards[2]. In this process of matching, the intensity of each peak is treated differently based on the ion identity. MS/MS ions are clustered into three types: "peripheral-cut" ions, formed by neutral loss of water, $CO_2$, amino acid, or amines derived from functional groups linking to LM carbon-chain as hydroxy, hydroperoxy, carbonyl, epoxy, carboxy, amino acid group, or amino group; "chain-cut" ions, formed by cleavage of a carbon-carbon bond along the LM carbon-chain; and "chain-plus-peripheral-cut" ions, formed by combination of chain-cut and peripheral-cut. Molecular ions formed during electrospray(ESI) can be converted easily to peripheral-cut ions in the MS/MS process. Similarly, chain-cut ions can also be converted readily to chain-plus-peripheral-cut ions (Scheme 4).

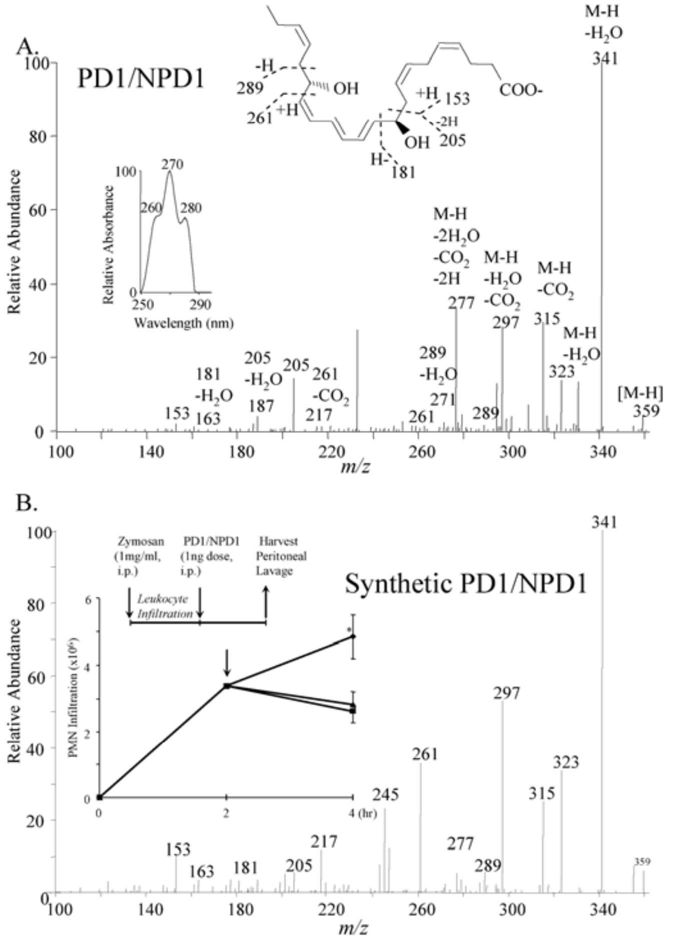

**FIGURE 5.** Mass spectra of biogenic and synthetic PD1/NPD1. (A) MS/MS spectrum of PD1/NPD1 generated in trout brain cells[38]. (B) MS/MS spectrum of synthetic PD1 with inset showing that PD1 treatment during the course of acute inflammation halts PMN infiltration (* $p < 0.05$) induced by zymosan[39].

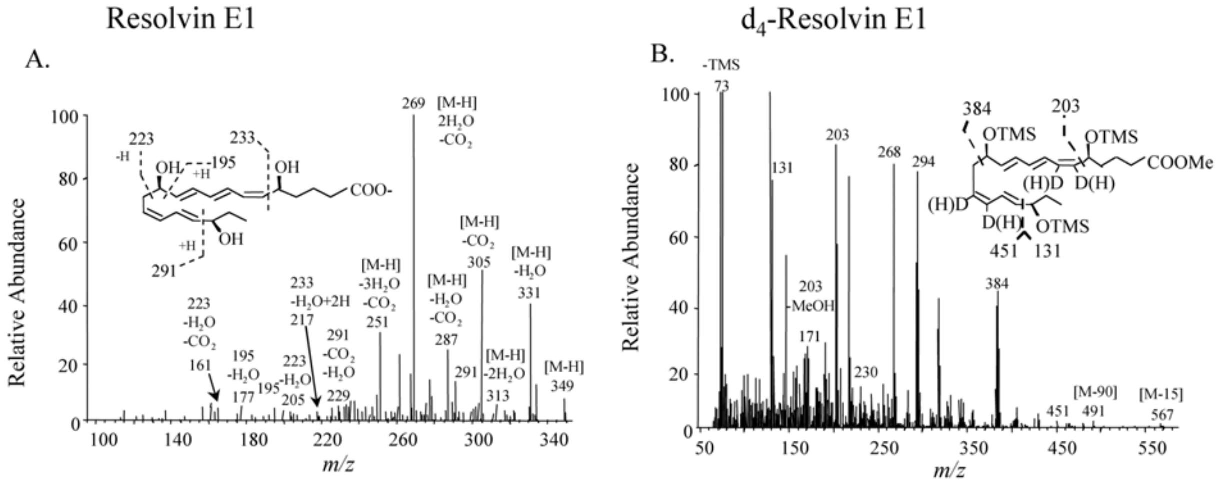

**FIGURE 6.** MS/MS spectrum of synthetic RvE1 and GC-MS spectrum of deuterated-RvE1. (A) MS/MS spectrum of synthetic RvE1. (B) GC-MS spectrum of the deuterated-RvE1 with methylation and silylation.

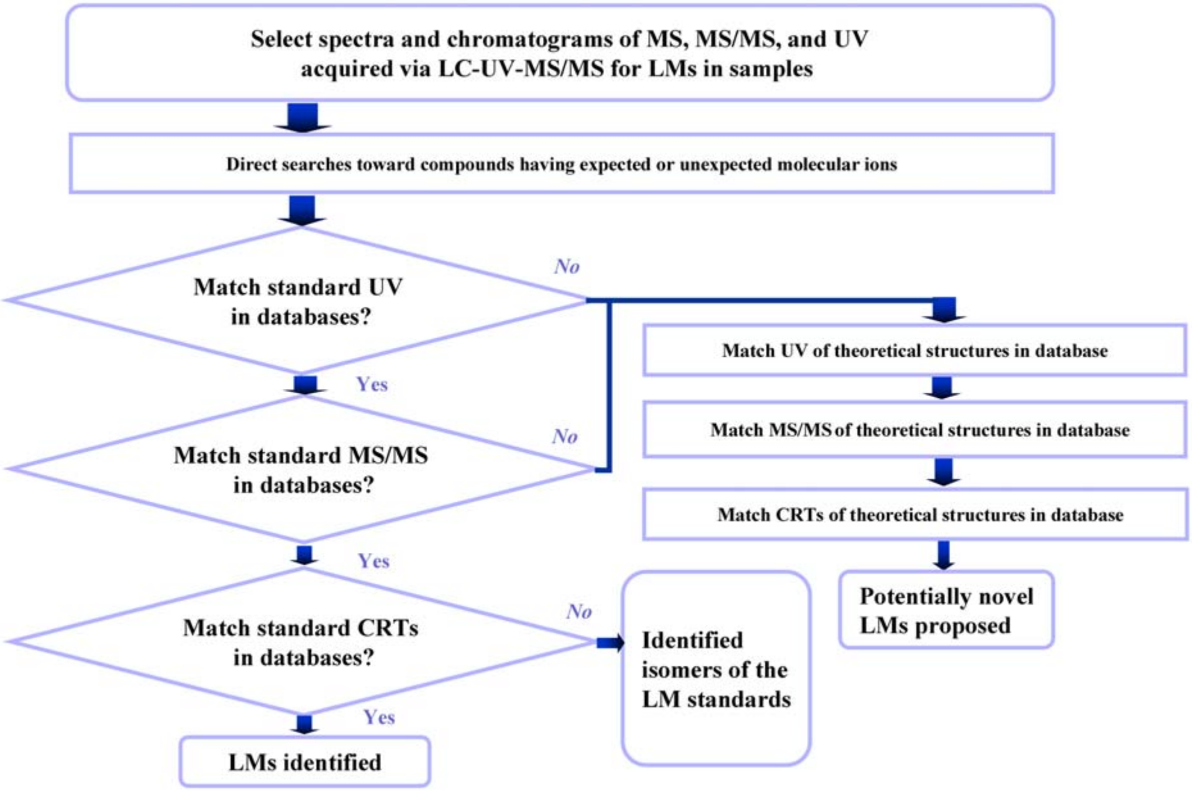

**SCHEME 3.** Logic diagram for developing LC-UV-MS/MS-based mediator databases and search algorithms for lipidomic analyses of PUFA-derived LMs[2].

Typical chain-cut ions for LMs in MS/MS are formed by α-cleavage of the carbon-carbon bonds connecting to the carbon with a functional group directly attached[4,6,8,13,25]. LMs readily undergo α-cleavage[6]. We proposed the nomenclatures illustrated in the $LXA_4$ structure presented in Scheme 4 to systematically name the segments formed via chain-cut and chain-plus-peripheral-cut without concern for hydrogen-shift occurring during mass spectrometric analysis of PUFA-derived products. All the possible chain-cut, peripheral-cut, and chain-plus-peripheral-cut segments for $LXA_4$ are indicated; the details for nomenclatures can be found in Lu et al.[2].

A MS/MS ion detected from LM samples in negative-ion mode generally is formed from a specific segment with the addition or subtraction of hydrogen(s) caused by hydrogen-shift during the cleavage[2]. The charge (z) of the LM negative ion is usually equal to one; therefore, the mass-to-charge ratio (*m/z*) of a LM ion is usually equal to its mass (m). Previous reports[4,6] and our published results[2,3,17,20] demonstrated that the MS/MS fragmentation observes the empirical rules on the addition or subtraction of hydrogen(s) for the chain-cut segments to form the chain-cut MS/MS ions[see reference 2 and scheme 4 for example].

- For the segment Cc, the detected MS/MS ions are Cc, Cc + H.
- For the segment Cm, the detected MS/MS ions are Cm, Cm ± H, and Cm ± 2H.
- For the segment Mc, the detected MS/MS ions are Mc and Mc – H.
- For the segment Mm, the detected MS/MS ions are Mm, Mm ± H, and Mm ± 2H.

These represent the general rules applied to each compound and were used to identify the segments for instrument-detected MS/MS ions. The interpreted ions, such as M - H - $H_2O$, Cc, Cc + H, or Mc – H, identified as the MS/MS ions detected in the mass spectrometer, are called virtual ions. One detected

MS/MS ion can be interpreted as one or several virtual ions. Through loss of $H_2O$, $CO_2$, $NH_3$, and/or amino acids, the chain-cut ions can form chain-plus-peripheral-cut ions. For the chain-cut and chain-plus-peripheral-cut ions in the present report, we focused on those formed by α-cleavages. Those detected MS/MS ions uninterpretable via the empirical rules mentioned above and neutral loss are taken as unidentified ions.

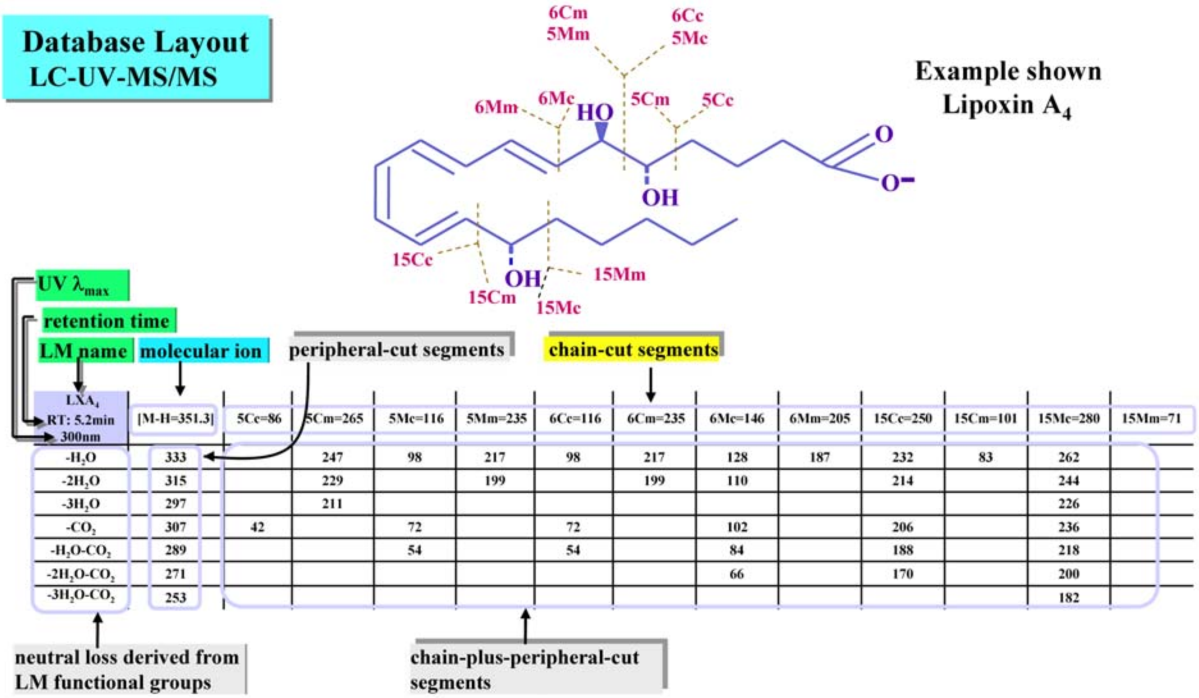

**SCHEME 4.** LC-UV-MS/MS database layout: example for naming LM segments[2]. In this case, example shown is lipoxin $A_4$, formed via chain-cut, peripheral-cut, and chain-plus-peripheral-cut for interpretation of MS/MS fragmentation.

## *Modification of MS/MS Ion Intensities According to Identities*

Chain-cut ions are most informative and could be diagnostic for determining specific LM structures such as the position of functional groups and double bonds. Peripheral-cut ions in MS/MS spectra are similar among LM isomers and, therefore, were not specific enough for differentiation of individual LM isomers[2].

According to empirical fragmentation rules mentioned above, the $n^{th}$ MS/MS peak can be identified as one or several chain-cut (*C*) ions, peripheral-cut (*P*) ions, and/or chain-plus-peripheral-cut (*CP*) ions. The weighted intensity ${}^{y}I_n$ of each identified ion is as follows:

$$ {}^{y}I_n = I_n' \div \left( {}^{C}_{n}M + {}^{CP}_{n}M + {}^{P}_{n}M \times \rho \right) \times {}^{y}W \ldots(a) $$

where:

*y* is the MS/MS ion type identified as *C*, *P*, or *CP*;

$I_n'$ is the relative **i**ntensity of the **n**$^{th}$ peak in the MS/MS spectrum;

$^{c}_{n}$M is the number of **c**hain-cut ions identified for the **n**th MS/MS peak;

$^{cp}_{n}$M is the number of **c**hain-plus-**p**eripheral-cut ions identified for the **n**th MS/MS peak;

$^{p}_{n}$M is the number of **p**eripheral-cut ions identified for the **n**th MS/MS peak; and

$^{y}$W is the **w**eight measuring the importance of the identified ion to determine the LM structure.

It is 10 as $^{C}$W and 1 as$^{CP}$W or $^{P}$W (for peripheral-cut ions). The fingerprint features of chain-cut ions are used to define LM structure by multiplying their intensities by 10, which was determined to be the best among values 2, 10, 20, and 100 tested. Weighted MS/MS ion intensities are used for COCAD and the theoretical system. ρ represents the contribution of peripheral-cut ions to $I_n'$ (ρ = 3 for peripheral-cut ions formed via loss of one $CO_2$ from each molecular ion, ρ = 10 for peripheral-cut ions formed via loss of one $H_2O$ from each molecular ion, and ρ = 1 for other peripheral-cut ions formed via multiple loss of $CO_2$ and/or $H_2O$ from each molecular ion). The assignment of ρ values is arbitrary and based on the observation of relative intensities of peripheral-cut ions in MS/MS spectra of LMs.

### COCAD Contrast Angle

COCAD used a contrast-angle algorithm to match an MS/MS spectrum between sample and standards. For this approach, the contrast angle is calculated as follows:

$$C_v = \sum_{n=1}^{N} ({}^{C}_{n}B_v \times {}^{C}I_n) \qquad \text{…(b)}$$

$$CP_v = \sum_{n=1}^{N} ({}^{CP}_{n}B_v \times {}^{CP}I_n) \qquad \text{…(c)}$$

$$P_v = \sum_{n=1}^{N} ({}^{P}_{n}B_v \times {}^{P}I_n) \qquad \text{…(d)}$$

$$D_C\ (D_{CP,}\text{ or } D_P) = \frac{\sum_{v=1}^{V} U_v S_v}{\sqrt{\sum_{v=1}^{V} U_v^{\,2} \sum_{v=1}^{V} S_v^{\,2}}} \qquad \text{…(e)}$$

$U_v$ is equal to $C_v$, $CP_v$, or $P_v$ for unknown spectrum to be identified;

$S_v$ is equal to $C_v$, $CP_v$, or $P_v$ for standard spectrum.

$$\text{COCAD contrast angle} = \text{Cos}^{-1[}(10 \times D_C + D_P + D_{CP}) \div (11 + \omega_{CP})] \qquad \text{…(f)}$$

where:

ν is the ν$^{th}$ virtual ion;

*V* is the total number for one type of virtual ion formed via chain-cut, chain-plus-peripheral-cut, or peripheral-cut for a specific LM;

${}^{C}_{n}B_{v}$ is equal to 1 if the n^th MS/MS peak can be identified as the $\nu^{th}$ virtual ion formed via chain-cut, or equal to zero if not;

${}^{CP}_{n}B_{v}$ or ${}^{P}_{n}B_{v}$ has a similar meaning but for ions formed via chain-plus-peripheral-cut or peripheral-cut;

N is the total number of peaks in the MS/MS spectrum;

$D_C$ is the dot product between the virtual vectors of *U* (unknown sample) and

*S* (standard) formed via chain-cut;

$D_{CP}$ or $D_P$ is the dot product for chain-plus-peripheral-cut or peripheral-cut ions, respectively.

$D_C$, $D_{CP}$, or $D_P$ in (e) represents the similarity of ions formed via chain-cut, chain-plus-peripheral-cut, or peripheral-cut, between an unknown spectrum and a standard spectrum. None of them is greater than 1. The $\nu^{th}$ virtual ion is not used for the calculation of the corresponding $D_C$, $D_{CP,}$ or $D_P$ if either $U_v$ or $S_v$ is zero. If every $C_v$, $CP_v$, or $P_v$ within the vectors is zero, then $D_C$, $D_{CP,}$ or $D_P$ is assigned the value zero, respectively.

The COCAD contrast angle in formula (f) represents how well the spectrum of the sample matches the standard: if it is $0^o$, the two spectra match exactly; if it is $90^o$, the two spectra do not match at all; the smaller the contrast angle between $0^o$ and $90^o$, the better the match[24,26]. The value is integrated and normalized from dot products $D_C$, $D_{CP}$, and $D_P$ (f). The numeric coefficient 10 in (f) was found to be the best value (2, 20, and 100 were also tested) that emphasizes the fingerprinting feature of chain-cut ions because chain-cut ions are more important for determining the LM structure than are other types of ions. To normalize$[(10 \times D_C + D_{CP} + D_P) \div (11 + \omega_{CP})]$ in (f) to be no more than 1, 11 was used in the denominator of (f), and $\omega_{CP}$ is equal to 1 if at least one MS/MS ion is identified as a chain-plus-peripheral-cut virtual ion or equal to zero if no such ion is identified. No chain-plus-peripheral-cut ion is identified in a few LM standard spectra. Therefore, $\omega_{CP}$ is introduced in equation (f) to normalize the COCAD contrast angle to zero when matching these types of spectra against themselves. Unidentified ions were excluded for matching in equations (b) to (f).

According to the empirical fragmentation rules, for mono-hydroxy-containing LMs, the $\nu^{th}$ chain-cut ion can be Cc, Cc+1, Cm-2, Cm-1, Cm, Cm+1, Cm+2, Mc-1, Mc, Mm-2, Mm-1, Mm, Mm+1, or Mm+2. The $\nu^{th}$ chain-plus-peripheral-cut ion is Cc-$CO_2$, Cc-$CO_2$+1, Cm-$H_2O$-2, Cm-$H_2O$-1, Cm-$H_2O$, Cm-$H_2O$+1, Cm-$H_2O$+2, Mc-$H_2O$-1, Mc-$H_2O$, Mc-$CO_2$-1, Mc-$CO_2$, Mc-$H_2O$-$CO_2$-1, or Mc-$H_2O$-$CO_2$. The $\nu^{th}$ peripheral-cut ion is M-H-$CO_2$, M-H-$H_2O$, or M-H-$H_2O$-$CO_2$. For LMs having multiple functional groups, *V* is accordingly greater.

## Theoretical Database and Search Algorithm for the Identification of Novel Lipid Mediators

Theoretical databases consist of the segments (Scheme 4), the UV $\lambda_{max}$, and RTs predicted for potentially novel LMs. Searching against a theoretical database is also performed stepwise as described in Scheme 3, from UV $\lambda_{max}$, to MS/MS spectra, and then to RTs.

Equation (g) is the matching score for an MS/MS spectrum of an unknown product compared with a virtual spectrum based on the segments and empirical fragmentation rules noted above.

$$\text{Matching score} = \{\sum_{f=1}^{F} [\sum_{n=1}^{N} ({}^{C}\mathrm{I}_{n} \times {}^{C}_{f}\mathrm{M}_{n}) \times ({}^{f}\mathrm{T}_{C} \div {}^{f}\mathrm{A}_{C})^{0.5}]$$

$$+ \sum_{f=1}^{F} [\sum_{n=1}^{N} ({}^{CP}\mathrm{I}_{n} \times {}^{CP}_{f}\mathrm{M}_{n}) \times ({}^{f}\mathrm{T}_{CP} \div {}^{f}\mathrm{A}_{CP})^{0.5}] + \sum_{n=1}^{N} [{}^{P}\mathrm{I}_{n} \times {}^{P}\mathrm{M}_{n} \times (\mathrm{T}_{P} \div \mathrm{A}_{P})^{0.5})]\}$$

$$\div \sum_{n=1}^{N} ({}^{C}\mathrm{I}_{n} + {}^{CP}\mathrm{I}_{n} + {}^{P}\mathrm{I}_{n}) \qquad \text{(g)}$$

The matching score in (g) summates the weighted intensities of all the identified MS/MS peaks in the spectrum acquired from the sample. The numerator of the formula is composed of three parts:

$$\sum_{f=1}^{F} [\sum_{n=1}^{N} ({}^{C}\mathrm{I}_{n} \times {}^{C}_{f}\mathrm{M}_{n}) \times ({}^{f}\mathrm{T}_{C} \div {}^{f}\mathrm{A}_{C})^{0.5}]$$

summating the weighted intensities of MS/MS peaks identified as chain-cut ions;

$$\sum_{f=1}^{F} [\sum_{n=1}^{N} ({}^{CP}\mathrm{I}_{n} \times {}^{CP}_{f}\mathrm{M}_{n}) \times ({}^{f}\mathrm{T}_{CP} \div {}^{f}\mathrm{A}_{CP})^{0.5}]$$

summating the weighted intensities of MS/MS peaks identified as the chain-plus-peripheral-cut ions; and

$$\sum_{n=1}^{N} [{}^{P}\mathrm{I}_{n} \times {}^{P}\mathrm{M}_{n} \times (\mathrm{T}_{P} \div \mathrm{A}_{P})^{0.5})]$$

summating the weighted intensities of MS/MS peaks identified as the peripheral-cut ions. ${}^{C}_{f}\mathrm{M}_{n}$ is the total number of chain-cut ions via α-cleavage formed from the $f^{th}$ functional group and matched to the $n^{th}$ MS/MS peak. F is the total number of functional groups in one LM. f is counted from the carboxyl terminus of LM. For example, f is 1 for 5-hydroxy, 2 for 6-hydroxy, and 3 for the 15*S*-hydroxy group present in $LXA_4$. F for $LXA_4$ is 3 (Scheme 4).

$$\sum_{n=1}^{N} ({}^{C}\mathrm{I}_{n} \times {}^{C}_{f}\mathrm{M}_{n})$$

summates the weighted intensities of MS/MS peaks identified as chain-cut ions formed from the $f^{th}$ functional group via α-cleavage. The smallest MS/MS ion detectable in ion-trap MS/MS is generally ~ *m/z* 95 for LMs with a molecular ion of ~400 Da[27]. To compensate for the bias caused by the inability to detect an MS/MS ion of *m/z* less than 95, factors $({}^{f}\mathrm{T}_{C} \div {}^{f}\mathrm{A}_{C})^{0.5}$, $({}^{f}\mathrm{T}_{CP} \div {}^{f}\mathrm{A}_{CP})^{0.5}$, and $(\mathrm{T}_{P} \div \mathrm{A}_{P})^{0.5}$ are used in (g). ${}^{f}\mathrm{T}_{C}$ (or ${}^{f}\mathrm{T}_{CP}$) is the total number of chain-cut (or chain-plus-peripheral-cut) ions formed from the $f^{th}$ functional group via α-cleavage. $\mathrm{T}_{p}$ is the total number of peripheral-cut ions formed from one LM. ${}^{f}\mathrm{A}_{C}$ (${}^{f}\mathrm{A}_{CP}$, or $\mathrm{A}_{P}$) is the fraction of ${}^{f}\mathrm{T}_{C}$ (${}^{f}\mathrm{T}_{CP}$, or $\mathrm{T}_{P}$) representing the ions within the MS/MS detecting range (*m/z* from 95 to the *m/z* of molecular ion). 20-HETE is a typical example. The ions formed from the segment 20Cm and 20Cm–$H_2O$ of 20-HETE are *m/z* 31 and 13 according to empirical fragmentation rules, which are too small to be detected in ion-trap MS/MS. Consequently, without the use of factors (${}^{f}\mathrm{T}_{C}$

$\div\, {}^{f}A_C)^{0.5}$ and $({}^{f}T_{CP} \div {}^{f}A_{CP})^{0.5}$, 20-HETE could have a lower matching score than other HETEs even though the MS/MS spectrum is that of 20-HETE.

$$\sum_{n=1}^{N} ({}^{C}I_n + {}^{CP}I_n + {}^{P}I_n)$$

is used in (g) for normalization to eliminate the impact on the matching scores of the total peak intensities in MS/MS spectra.

The databases and search algorithms were developed on the basis of LC-UV-ion trap MS/MS data of LMs. The ion intensity patterns of MS/MS spectra generated from an ESI-triple quadrupole mass spectrometer are quite similar to ESI-ion trap MS because the collision energy for both types of instruments is in the low energy region (a few to 100 eV, laboratory kinetic energy of ions)[6,21,28,29]. Therefore, the constants and algorithms reviewed here and in Lu et al.[2] may fit the CID spectra from triple-quadrupole MS/MS without much modification. For high-collision-energy (several $10^2$ to ~$10^3$ eV) CID spectra generated via sector or TOF/TOF analyzer, the relative intensity patterns are quite different in comparison with the low-energy ones, although many ions occurred for both energy situations[4,6,21,28,29]; for example, the peripheral-cut ions are less abundant than the chain-cut ions. For ion-trap and triple-quadrupole MS/MS, peripheral-cut ions are more abundant than chain-cut ions. Our constants and algorithms give chain-cut ions more weight than peripheral-cut ions because chain-cut ions are more important to define LM structures. Therefore, they may still fit high-collision-energy CID spectra. Nevertheless, the constants and algorithms should be thoroughly tested and modified accordingly to fit other instruments that may generate different fragmentation patterns and intensities of resulting ions.

The RTs used in this set of experiments were obtained using specified chromatographic conditions (for a column of 100-mm length and some for 150-mm length) and because several fundamental issues were our initial focus. Hence, these databases and algorithms were programmed so that the new LC-UV-MS/MS data including other chromatographic conditions can be easily entered and used in the databases.

A computer-based automated system equipped with these databases and searching algorithms was used successfully to identify 15*S*-HETE in murine spleen[2] (Fig. 7). The peak displayed at RT 20.4 min on the chromatogram (at *m/z* 219 of MS/MS 319, left inset) had a UV $\lambda_{max}$ 235 nm. Therefore, the search on the theoretical system was narrowed down to the subdatabase with molecular ion *m/z* 319, UV $\lambda_{max}$ 235 nm, and RT 21 min. In this case, 15-HETE gave the highest matching score among all compounds in the subdatabase. The MS/MS peaks identified were annotated with the ion interpretation that also shows a fragmentation mechanism[2]. Segments of 15-HETE that matched the MS/MS peaks according to the empirical fragmentation rules are italicized[2]. MassFrontier™ was also used to identify the peak, which also identified it as 15-HETE[2].

## ELISA-Based Lipidomic Analysis and Physical Validation

Specific ELISA for the AT $LXA_4$ (ATL or 15-epi-$LXA_4$) with high selectivity (cross-reactivity to $LXA_4$ is less than 3%) and sensitivity (detection limit is 50 pg/ml) were developed[10,30]. Using this specific ELISA, we demonstrated that aspirin therapy triggers the production of anti-inflammatory ATL in healthy individuals in an 8-week, randomized and placebo-controlled clinical trial. ATL production in the test groups was inversely related to inhibition of platelet thromboxane, even when aspirin was given in low doses (81 mg of aspirin daily). Thus, utilizing this specific ELISA, we are able to monitor plasma ATL providing an easy tool and positive signal for assessing individual responses to aspirin therapy. All ELISAs will be validated for each series of LMs using LC-MS/MS as in Chiang et al.[10].

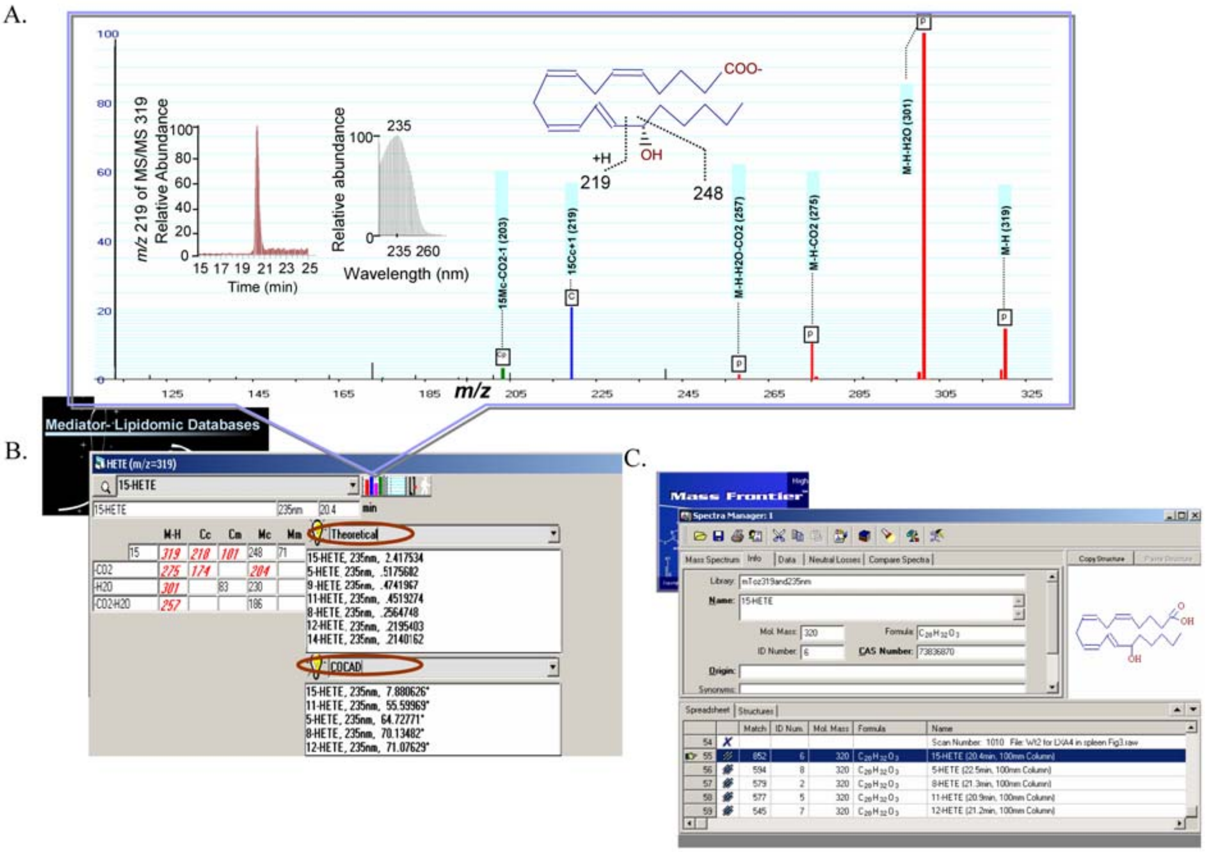

**FIGURE 7.** Using a computer-based automated system equipped with databases and newly devised searching algorithms developed in this lab indicated the presence of 15*S*-HETE in murine spleen[2]. (A) Identification and interpretation of anions in a MS/MS (at *m/z* 319) spectrum. The MS/MS scan conditions were: parent ion, *m/z* 319; isolation width, 1.5; normalized collision energy, 42%; activation time, 30 ms; and scan of MS/MS ions, from *m/z* 95–323. C, chain-cut ion; CP, chain-plus-peripheral-cut ion; and P, peripheral-cut ion. Insets are for identified 15*S*-HETE in the sample: *left*, chromatogram (at *m/z* 219 of MS/MS 319), which was the intensity temporal profile or selective ion chromatogram at *m/z* 219 in MS/MS spectrum of parent ion *m/z* 319 generated in ESI; *middle*, UV spectrum showing maximal absorption at 235 nm; *right*, the proposed MS/MS segments on 15*S*-HETE. The LC column was 100 mm long. (B) Report for the search via the Theoretical system shows 15-HETE has the highest matching score; report for the search via COCAD shows 15-HETE has the smallest contrast angle. (C) 15-HETE also had the highest matching score when the LC-UV-MS/MS data were searched against the database built with MassFrontier$^{TM}$.

## PROTEOMIC ANALYSIS OF RESOLVING EXUDATES

### 2D Gel Electrophoresis

Soluble proteins from biomedical samples will be separated by isoelectric focusing and SDS-PAGE, according to[31,32]. Proteins are solubilized in a total volume of 185-µl rehydration buffer of the following composition: 7 *M* urea, 2 *M* thiourea, 4% CHAPS, 30 m*M* dithiothreitol (DTT), 0.2% v/v ampholytes (BioLyte pH 3-10, Bio-Rad, CA), 0.001% bromophenol blue. Isoelectric focusing strips with a linear immobilized pH gradient ranging from 3–10 (Bio-Rad) are rehydrated with sample-containing rehydration buffer for 30 min in the isoelectric focusing tray, overlaid with mineral oil, and further rehydrated actively at 50 V for 16 h in a Protean isoelectric focusing apparatus (Bio-Rad). Isoelectric focusing is subsequently performed by increasing the voltage linearly over 20 min to 250 V, followed by a linear increase over 2.5 h to 8,000 V and further focusing at 25,000 V-h at 8,000 V. The focused proteins are reduced by DTT for 10 min at room temperature in equilibration buffer 1, composed of 6 *M*

urea, 2% SDS, 0.375 *M* Tris/HCl pH 8.8, 20% glycerol, and 130 m*M* DTT, followed by thioether formation by iodoacetamide for 10 min at room temperature in the dark in equilibration buffer 2 composed of 6 *M* urea, 2% SDS, 0.375 *M* Tris/HCl pH 8.8, 20% glycerol, and 135 m*M* iodoacetamide. The IPG strips are rinsed once in SDS-PAGE running buffer (25 m*M* Tris, 0.19 *M* glycine, 3.5 m*M* SDS), and mounted in agarose on top of 10.5–14% gradient SDS-polyacrylamide gels (Bio-Rad). Proteins are separated by size (range ~15–200 kDa) via electrophoresis with running buffer at 200 V for 60 min at 19$^o$C in a Dodeca tank (Bio-Rad), which allows multiple gels to be run.

## In-Gel Protein Digestion and Peptide Recovery

The gels are fixed and stained with Sypro Ruby protein gel stain[33]. Protein gel spots of interest are excised and cut in ~1-mm$^3$ cubes with clean scalpels and placed in Eppendorf tubes. The protein is in-gel digested with trypsin, and peptides are recovered essentially as described by Rosenfeld et al.[34], with the following modifications: the gel pieces are washed twice for 45 min in 0.5 *M* Tris pH 9.2/acetonitrile 1:1 (v/v) at 37$^o$C. They are shrunk in acetonitrile and dried for 10 min by vacuum centrifugation (SpeedVac, Savant, NY). They are swollen in 100 m*M* $NH_4HCO_3$ for 30 min at room temperature. They are shrunk again in acetonitrile and dried. In-gel trypsin digestion is performed by the addition of 100 µl of 50 m*M* $NH_4HCO_3$ containing 500 ng of modified sequencing-grade trypsin (Promega, WI). After 1 h, additional 50 m*M* $NH_4HCO_3$ is added to just cover the gel pieces. The in-gel digestion proceeds overnight at 28$^o$C. The fluid surrounding the gel pieces is transferred to a clean Eppendorf tube. The gel pieces are incubated for 1 h with 5% formic acid in 50% acetonitrile, and the extract is pooled with the first supernatant. The gel pieces are incubated another 15 min with 100% acetonitrile, which is transferred to the pooled peptide extracts. The combined extracts are dried by vacuum centrifugation (SpeedVac), and the tryptic digests are stored at -80$^o$C until further analysis.

## LC-Nanospray-MS/MS Proteomic Analysis

Tryptic peptide mass and charge will be determined by capillary LC/electrospray ionization ion-trap MS/MS[35,36]. Tryptic peptides are separated by capillary LC using a capillary column (LC Packings, ID 75 µm, length 15 cm, particle size 3 µm) at 100 nl/min delivered by an Agilent 1100LC pump (400 µl/min) and a flow splitter (LC Packings, Accurate, NY). Peptides are loaded via a Rheodyne port onto a 2-µg capacity peptide trap (CapTrap, Michrom, CA) in 2% acetonitrile, 0.1% formic acid, and 0.005% trifluoroacetic acid. A mobile-phase gradient is run using mobile phase A (2% acetonitrile and 0.1% formic acid in ultrapure water) and B (80% acetonitrile and 0.1% formic acid in ultrapure water) from 0–10 min 0–20% B, 10–90 min 20–60% B. Water and acetonitrile are of mass spectral grade (Burdick & Jackson, MI). Peptide mass and charge are determined after low-flow electrospray ionization on a ThermoFinnigan Advantage ion-trap mass spectrometer. Electrospray ionization is performed with end-coated spray tips (silica-tip 5 cm, ID 360 µm, tip ID 15 µm, New Objective, MA) at a final flow rate of approximately 100 nl/min and a spray voltage of 1.8 kV. The mass spectrometer is tuned with angiotensin II in 30% B at 100 nl/min. Peptide parent ion mass is determined, and zoom scans and tandem MS/MS spectra of parent peptide ions above a signal threshold of $2 \times 10^4$ are recorded with dynamic exclusion using Xcalibur v. 1.3 data acquisition software (ThermoFinnigan).

## Peptide Mapping

Protein identification of gel spots will be made by peptide mapping of tryptic peptide tandem mass spectra using Sequest. Sequest searches are performed within the BioWorks 3.1 software (ThermoFinnigan), using the NCBI nr.fasta protein database indexed for mouse proteins. Possible protein modifications taken into consideration will include alkylation of cysteine with iodoacetamide and acrylamide and oxidation of methionine. A protein is considered positively identified if a minimum of two tryptic peptides of that protein are matched with a cross-correlation score above 2.0.

An example of determining the temporal changes of specific exudate proteins is shown in Fig. 8. We used a MS-based proteomic analysis with 2D gel electrophoresis and image analysis. Proteins were identified by peptide mapping of in-gel-digested proteins using capillary LC-nanospray ion trap MS/MS (nanospray-LC-MS/MS) and bioinformatics software. Fig. 8 shows a representative 2D gel of exudate proteins and the temporal profiles of several proteins with distinct kinetics during inflammation resolution. A list of proteins and their corresponding identified tryptic peptide fragments together with cross-correlation scores can be found in Bannenberg et al.[37], as well as the observed and theoretical (m.w.) and isoelectric point (pI) of the identified proteins. Serum proteins such as plasminogen, fibrinogen, and serum albumin were abundant in exudates 4 h after initiation of inflammation, indicating that protein exudation from blood made the largest contribution to the total exudate protein levels[37]. Haptoglobin (Fig. 8, B and C) displayed a delayed accumulation that is maximal at the onset of resolution interval(Ri). S100A9 rapidly accumulated in the exudate, achieving maximal levels during Ri, followed by a gradual decrease at 24 h. The exudate level of a C-terminal fragment of $\alpha_1$-macroglobulin (pregnancy zone protein), plasminogen, and fibrinogen displayed the same kinetics as the total exudate protein levels[37]. In contrast, apolipoprotein E was present in the uninflamed peritoneum; its levels decreased during the Ri and returned to basal levels after 24 h. Proteinase inhibitor 1a rapidly appeared in the peritoneal exudate, with maximal levels at 4 h that thereafter decreased continuously. Transthyretin levels apparently did not change during the time course (Fig. 8B). Using this approach of “resolution proteomics”, we identified several components that are likely founding members of the resolvers in novel resolution circuits and pathways operating *in vivo* during and promoting resolution.

## SUMMARY

LM informatics and proteomics applied in inflammation research and specifically to mapping the resolution phase provides a powerful means of uncovering specific biomarkers for potential disease phenotypes. By incorporating them with system biology analysis, we can begin to elucidate relationships among changes across a wide range of biomolecule classes and provide new insight into the pathophysiology of inflammatory disease.

LM informatics employing LC-UV-MS/MS to evaluate and profile temporal production of compounds, combined with proteomics at defined points during experimental inflammation and its resolution, enable us to elucidate bioactions and roles of novel mediators in inflammation and resolution. The automated system reviewed here including databases and searching algorithms is crucial for prompt and accurate analysis of these lipid mediators such as eicosanoids, resolvins, and protectins, which play critical roles in human physiology and many prevalent diseases, especially those related to inflammation.

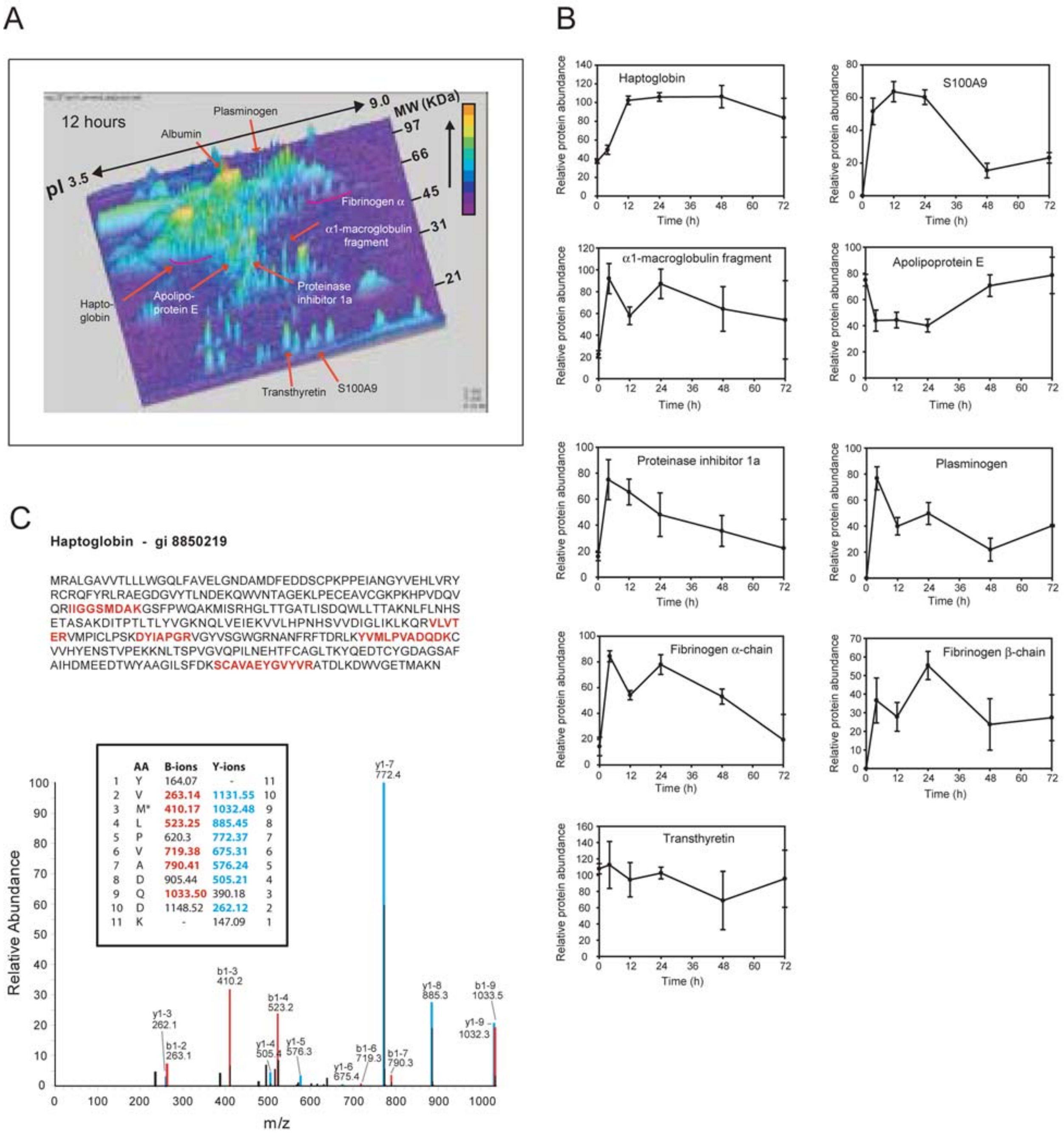

**FIGURE 8.** Proteomic temporal analysis of exudate proteins[37]. (A) Mice were injected with zymosan A to induce peritonitis. Exudate fluid was collected at the indicated time points and proteins were separated by 2D gel electrophoresis. Changes in individual protein levels were measured by image analysis. Selected proteins that display temporal regulation are indicated (arrows) and were identified by LC-MS/MS and peptide mapping. (B) The temporal profiles of several exudate proteins (haptoglobin, S100A9, a C-terminal fragment of $\alpha_1$-macroglobulin, apolipoprotein E, proteinase inhibitor 1$\alpha$, plasminogen, the fibrinogen $\alpha$- and $\beta$–chains, and transthyretin) are shown (values are means ± SEM, n = 3–6 gels). (C) Tryptic peptide mapping of haptoglobin by MS. Peptides that are matched are shown in red. The matching of the tandem mass spectrum of peptide YVMLPVADQDK is shown.

## ACKNOWLEDGMENTS

Many thanks to Mary Halm Small for expert editorial assistance in manuscript preparation and Katherine Percarpio for lab assistance. We thank Nicos A. Petasis (University of Southern California) and Core C of NIH grant no. P50-DE016191 for preparation of synthetic $d_4$-Resolvin E1. This work was supported in part by grants no. GM38765, DK074448, and P50-DE016191 (S.H., Y.L., C.N.S.) from the National Institutes of Health.

### Terms and Abbreviations Used in this Report

| | |
|---|---|
| 2D | Two dimensional |
| 5S,15S-diHETE | 5S,15S-dihydroxy-eicosa-6E,8Z,10Z,13E-tetraenoic acid |
| 5S-HETE | 5S-hydroxy-eicosa-6E,8Z,11Z,14Z-tetraenoic acid |
| 11S-HETE | 11S-hydroxy-eicosa-5Z,8Z,12E,14Z-tetraenoic acid |
| 12S-HETE | 12S-hydroxy-eicosa-5Z,8Z,10E,14Z-tetraenoic acid |
| 15S-HETE | 15S-hydroxy-eicosa-5Z,8Z,11Z,13E-tetraenoic acid |
| 20-HETE | 20-hydroxy-eicosa-5Z,8Z,11Z,14Z-tetraenoic acid |
| AT | Aspirin triggered |
| AT-RvD1 | Aspirin-triggered-resolvin D1. (7S,8,17R-trihydroxy-docosa-4Z,9E,11E,13Z,15E,19Z-hexaenoic-acid) |
| AT-RvD2 | Aspirin-triggered-resolvin D2. (7S,16,17R-trihydroxy-docosa-4Z,8E,10Z,12E,14E,19Z-hexaenoic-acid) |
| AT-RvD3 | Aspirin-triggered-resolvin D3. (4S,11,17R- trihydroxy-docosa-5,7E,9E,13Z,15E,19Z-hexaenoic-acid) |
| AT-RvD4 | Aspirin-triggered-resolvin D4. (4S,5,17R-trihydroxy-docosa-6E,8E,10Z,13Z,15E,19Z-hexaenoic-acid) |
| AT-RvD5 | Aspirin-triggered-resolvin D5. (7S,17R-dihydroxy-docosa-5Z,8E,10Z,13Z,15E,19Z-hexaenoic-acid) |
| AT-RvD6 | Aspirin-triggered-resolvin D6. (4S,17R-dihydroxy-docosa-5E,7Z,10Z,13Z,15E,19Z-hexaenoic-acid) |
| CID | Collision-induced dissociation |
| COCAD | Cognoscitive-Contrast-angle Algorithm and Databases |
| DHA | Docosahexaenoic acid (C22:6) |
| DT | Docosatrienes |
| ELISA | Enzyme-linked immunosorbent assay |
| EPA | Eicosapentaenoic acid (C20:5) |
| GC-MS | Gas chromatography coupled with mass spectrometry |
| HETE | Hydroxy-eicosatetraenoic acid |
| IS | Internal standard |
| LC-UV-MS/MS | Liquid chromatography-ultraviolet spectrometry-tandem mass spectrometry |
| LMs | Lipid mediators |
| $LXA_4$ | Lipoxin $A_4$. (5S,6R,15S-trihydroxy-eicosa-7E,9E,11Z,13E-tetraenoic acid) |
| $LXB_4$ | Lipoxin $B_4$. (5S,14R,15S-trihydroxy-eicosa-6E,8Z,10E,12E-tetraenoic acid) |
| $LTB_4$ | Leukotriene $B_4$. (5S,12R-dihydroxy-eicosa-6Z,8E,10E,14Z-tetraenoic acid) |
| Nanospray | Nanoelectrospray ionization |
| PD1/NPD1 | Protectin D1/neuroprotectins D1 (10R,17S-dihydroxy-docosa-4Z,7Z,11E,13E,15Z,19Z-hexaenoic acid) |
| $PGB_2$ | 9-oxo-15S-hydroxy-prosta-5Z,8(12),13E-trien-1-oic acid |
| $PGE_2$ | 9-oxo-11α,15S-dihydroxy-prosta-5Z,13E-dien-1-oic acid |
| RT | Retention time |
| Rv | Resolvin |
| RvE1 | Resolvin E1 (5S,12R,18R-trihydroxy-eicosa-6Z,8E,10E,14Z,16E-pentaenoic acid) |
| SIM | Selective ion monitoring |
| SPE | Solid-phase extraction |
| ESI | Electrospray ionization |
| PUFA | Polyunsaturated fatty acids |
